# Address, Delivered before the New York Society of Dental Surgeons, by Its Retiring President, Dr. C. A. Marvin

**Published:** 1868-04

**Authors:** 


### DENTAL REGISTER.

Vol. XXII.] APRIL, 1868. [No. 4.

### Original Communications.

### ADDRESS

Delivered before the New York Society of Dental Surgeons, by its retiring President,

DR. C. A. MARVIN.

*Gentlemen of the New York Society of Dental Surgeons:*

In addition to the retiring address of the presiding officer, I proceed, in compliance with your expressed wish, to discharge the duty usually performed at the annual meeting of this Society, by some other member than the President. As you are aware, the appointment of an annual Essayist was made at the proper time, but the gentleman thus appointed, signified through the Secretary at our first meeting in February, that he would be unable to perform the expected service. The vote you then passed, devolved the duty upon me, and the preparation necessary for its performance has been made amid interruptions so numerous and under the pressure of engagements so weighty, that its completion in time for this evening seemed extremely problematical. The improvement of every spare interval of even a few minutes, has alone enabled me to redeem my pledge.

Twelve months ago, gentlemen, you called me to preside over this Society whenever it should convene, and to carry into effect as far as lay in my power, its rules and enactments.

Twelve months! What a period! Each month composed of a score and a half of days; each day, of a score and more of hours; each hour, of minutes three-score; each minute, of its three-score seconds; and each second of all these, sufficient in duration for the conception of a worthy thought, the formation of a wise resolution, the recognition of dependence upon the Supreme.

What an opportunity then for growth does a year afford; growth in the three directions of development just suggested, viz.: power of thought, strength of resolution and firmness of reliance upon the Infinite Father.

With this idea before us, it is becoming at this close of the year, to take a review of its spent months, its days and hours, all gone forever, and ask ourselves, "What has the year done for us?" And "what have we done for ourselves?"

The year now closed, has placed at our disposal, *time.*

When the period, whose review now occupies our attention, commenced, no human intelligence could foretell how much of it any one of us would be permitted to see. Whether the plans we then had formed, would have time afforded for their execution; whether the investigations in which we were then engaged, might be completed; or the experiments we then were testing, might be continued to their proof, was all unknown. In this uncertainty as to the duration of our existence, did we heed the voice of wisdom as it whispered,—

"Improve the present moment as it flies?"

Obeyed we her counsel? With renewed and persistent effort have we sought improvement? Have we applied every energy to the accomplishment of new and beneficial ends, that when the command—"Render thy account," shall come, we may not be found laggards, and wasteful of this precious gift,—*time?*

We condemn the man who, with capital placed in his hands for business purposes, allows it to lie useless and unproductive, and we have high authority for such judgment.

With us, time is capital, and if we fail to employ it so that some good accomplished shall stand as the result of its expenditure, we are culpable:—we have failed to execute our trust; we have slighted the gift and dishonored the giver.

The value of time must be appreciated at some period. If not while it is passing, it will be when it has passed. But the difference in the character of our appreciation of it before and after it has gone, is great. If valued aright while the possession of it is ours, it prompts to action, and a double good is effected:—the body, mind and conscience are developed healthily; and something is added to the great mass of things accomplished,—good done.

If on the other hand, the appreciation of time comes after it is gone, no feeling save that of remorse is experienced, and there is no feeling so terrible in its distressful character, as remorse.

The year just closed has given us twelve months of time. Have we rightly valued it? Have we well improved it? Is there anything added to our catalogue of achievements that was not found therein at the beginning of the year? These are questions which each can best answer for himself.

The time with which we have thus been favored, has gone forever. Whether valued or not, our possession of it has ended. Whether improved, or not, its record is completed. There will come a time for the *review* of that record, but for its *change*, never.

The year just closed has given us not only time but *opportunity* also.

Many a man has lived in obscurity because no opportunity of emerging therefrom, ever presented itself.

Many a man has burned with a desire to distinguish himself in some department of effort, in science, in letters, in art; but has gone to his grave unknown for want of opportunity. Time, he may have had, but no fortunate concurrence of circumstances constituting an opportunity. Scarcely can such a man be censured, for circumstances are imperious and few can control them.

But to a man chafing with desire to act, to carry into execution, plans, which he thinks will be productive of public good, to come in contact with his fellow men and secure their respect and praise, but against whose wishes, circumstances have ever arrayed themselves; to such a man, say, " Here is your opportunity, use it as you will," and you can give him no richer boon. You fill the measure of his earnest wish. With what eagerness he seizes the gift. How sincere his gratitude. How assiduous his application. How determined, how unintermitted his efforts to improve the occasion thus afforded. He values opportunity.

The man whose all engrossing thought is the acquirement of wealth, how eagerly he watches for that favorable juncture which is to furnish him, his opportunity.

The man who by the conspiracy of unfortunate circumstances, has come under the censure of his fellow men, with what avidity does he grasp the critical moment which is his opportunity to regain his fair reputation.

The man of science, whose whole soul is absorbed in study, investigation, experiment, to establish some long pondered theory; how narrowly he watches for the minute indication that accords with his expectation or hope, and how he clutches at it that he may make it the basis of a truthful demonstration.

To such men, opportunity is of vast importance. By such men, it is fully appreciated.

How is it with us? To the members of the New York Society of Dental Surgeons, there has been no lack of opportunity during the past year.

Its meetings have been held bi-weekly, and the door has ever stood invitingly open to all who desired to avail themselves of the opportunity for improvement therein afforded.

No gentleman has come here with a mind full of fresh thoughts, of valuable ideas and failed to utter them, but the fault has been his own. Freedom of utterance to all has been the distinguishing feature of our discussions.

Not only has there been a *willingness* to accord this privilege, but the Society, like a wise parent, solicitous for the highest welfare of those committed to his guardianship, has *urged* its members to avail themselves of it. It has not only said, "Here is your opportunity," but has added, "As you value what is of real worth, embrace the opportunity."

It is a characteristic of foolish human nature, to estimate but lightly that which is plentiful.

Our valuation of opportunities for improvement increases in exact proportion to their rarity and difficulty of acquirement.

Strange inconsistency of man! Ready to quarrel with the position in which such advantages are few or entirely wanting, but when placed in another position where they meet us in abundance, turning upon them an eye of cold indifference. Is not this the sublimity of folly?

Passing from a general to a specific and personal application of this department of our subject, I ask the question,—How many of *us* have rightly appreciated this rich gift of the year? How many have improved it? Not what proportion of membership of the Society, for the answer would not be to the credit of the Association. The attendance had been discreditably meagre.

But of those of us who have been present as regularly as has been consistent with our attention to other equally important duties, how many have valued aright our privileges?

Let each one ask himself the question,—Have I realized the fact that an opportunity passed, is gone forever? That if I have allowed it to go by unimproved, I have sustained just so much of positive loss,—a loss that can never be repaired?

Do we, each for himself, realize this truth? It is a truth, grave and undeniable. Each opportunity demands its own measure of improvement, and that measure is the measure of our entire ability. Nothing less will satisfy its demand, nothing more is required. No surplus of effort expended upon, or of fruit derived from, any one opportunity, can be placed to the credit of a former unimproved one. That has passed beyond our possession and gone to join the vast array of opportunities which await, in regular order of succession, the final review. Its record is closed. Nought can be added thereto, nought taken therefrom. There it lies in solemn silence, waiting, waiting. No voice of reproach comes from it now, no threat, no complaint of neglect. But bye and bye, it will rise to confront us, no longer voiceless nor passive. In tones that command attention, it will remind us of its sometime possession, of its store of good once ready for our gathering; of the doors of its treasury once inviting our entrance, but which we with criminal neglect allowed to remain closed between ourselves and the wealth within. Then, the review ended, with reproachful gaze it will add its record to the column of "opportunities lost," and glide away forever into the dead past.

How many of such abashing reckonings await us? How many such will be found within the twelve months of the closed year? Each one knows, and none beside save Him

"———— whose all pervading eye
Nought escapes without, within."

These may be called moral reflections. If they are, we shall not be harmed by allowing our minds to dwell upon them for a short interval.

Another view of this thought suggests the inquiry,—Can we afford to lose the benefit which such opportunities improved, would furnish? Can any degree of attainment short of the extremest possible measure, satisfy any right minded man? Certainly not.

The field of effort is wide, wider than a lifetime can cover. Heights of attainment there are, which tower far above our heads when we have exhausted our highest ability. The more we know, the more we perceive yet to learn. The realm of knowledge is infinite in extent, the capabilities of man are finite in power. This being so, how can we sit idly down and allow the opportunity for an advance to pass by without that advance being made?

Thus doing, *we defraud ourselves.* Do we not perceive it?

Thus doing, we allow others to pass us in the march and we are left in the rear ranks. Care we not for this?

Thus doing, we allow others to store their minds more and more with useful knowledge, while we add none to our limited supply.

Thus doing, we allow others to become more and more proficient in practical ability, while we make no progress.

Thus doing, we allow others to acquire freshness and vigor of thought, while our mental powers remain stagnant.

Is there one of us who can afford to be such a loser? The duties which devolve upon our profession, gentlemen, are laborious and intricate.

Dentistry has not as yet reached the development of maturity. It is just emerging from its infancy; and that it may grow and expand until it becomes that beneficent agency in the world that it is fitted and designed to become, and which *it will become*, it is important that all of its members should lend the aid of their zealous and persevering efforts.

There is no place in the ranks of Dental practitioners for laggards. They must seek elsewhere for a congenial element. Earnest, determined men bear aloft the banner of the profession, and as it unfolds to the breeze, the word inscribed thereon is seen of all. That word is "PROGRESS."

We who would participate in the honor of realizing to the profession the meaning of that word, must not move on reluctant foot nor neglect our opportunities. If we do, we lose all share in that honor. But, be it remembered, we lose no part of our responsibility. That still rests upon us with undiminished weight. And we must meet it, if not willingly, then coercively. And thus, every step of progress made by leading men in the profession, becomes matter of public knowledge, and just so much more is required of the Dental practitioner. If he have taken no part in the progressive movement, through disrelish of labor, he gains no exemption thereby, for now that more proficiency is expected of him, he must by increased exertion, come up to the required standard or confess himself inferior. Subterfuges will not long avail in a practical science.

If he deny the utility of the advance movement, he is soon confronted by the demonstration, and must prove it unsound or retire abashed. If he admit his want of knowledge of the recent improvements, he at once sinks in public estimation.

Is not the statement true then, that there is no room in the ranks of Dental practitioners for laggards? They either fall back themselves through indolence, or are thrust out by a discriminating public. And this is to be more and more the rule through every coming year. It *must* be, if our profession is to take its proper station. It *shall* be, if the efforts of devoted men can accomplish such a result.

I repeat the question then, can any one of us *afford* to neglect our opportunities for improvement? The answer is obvious.

One point further: the past year has furnished us in addition to time and opportunity, *means* also. This is of equal importance with those already considered.

A man may have time to perform a service, to secure a valuable end, to accomplish a worthy object, but no opportunity. He may have time and opportunity but no means. But to time and opportunity add means, and the trinity is complete. Henceforth the responsibility rests solely with him. Nothing can be added in the way of earthly advantages. If with these, he fail to accomplish something of good, he must lack either in ability or desire. If in the first, our mouths are closed and we leave him in the hands of Him who denied him ability; if in the second, we charge him with dishonoring his nature and his God.

How stands our account? Let us glance a moment at some of the means of improvement which the past year has brought us.

1st. The printed page to read. This is a most fruitful means of improvement when diligently used. Studying the writings of able men, and there are many such among the host of writers, strengthens the mind and arouses it into activity. If we meet therein occult ideas, whose meaning we do not at first view clearly perceive, reflection thereon until they are understood, has this double result; satisfaction that we have secured the idea, (if it be a good one) and quickened power of apprehension for succeeding ones.

Reading other's ideas elicits our own. No two minds take precisely the same view of things; that is, in every particular. Two men may advocate the same idea, but when detailing their reasons for so doing, one will certainly give some that the other does not, and omit some that the other gives. So when we read another's ideas on a certain subject, we form our own the while. They may agree in the main, but if so, our minds will not stop merely to agree. New thoughts will be originated, so that often one sentiment of the writer will be the text for a whole sermon of our own, the spark that fires a whole train of ideas that come flashing from the brain.

It is sufficient for the object in view, however, to notice only the primal benefit of reading, viz.;—gaining instruction. The reader reaps at a glance, the result of months, perhaps years of hard labor by the writer. It is a mistake to consider the labor of penning the article or book as the only labor its preparation has cost. The years of study and discipline necessary to fit the mind to generate such thoughts, and the culture needed to express them clearly and truthfully, must be taken into the estimate. One may utter in five minutes, truths which it has required a score of years to learn. When we hear the truths spoken, are we to credit their utterer only with the labor of speaking five minutes? If so, we defraud him out of the credit due for nineteen years, three hundred and sixty-four days, twenty-three hours and fifty-five minutes of hard mental labor!

The careful perusal of a well written work is the gathering of a harvest which another has planted and cultivated.

A second means of improvement furnished by the past year is the utterances of living men,—oral instruction. I t is no small privilege to listen to the words of men whose time and thought are expended for the advancement of science; whose earnestness of manner gives additional impressiveness to their words; whose living presence imparts a vitality to their instructions not otherwise possessed. Mind fires mind. The effect of one mind in full active operation, upon other minds, is electrical. Inspiration is communicated and received.

Here too, let me confine myself to the primal benefit,—instruction. A zealous laborer in the field of science, after hours of arduous application, comes before us, and in a few, well chosen, significant words, gives us the results of his investigations and experiments.

Then follows criticism. Questions sharply put and as readily answered, difficulties suggested and met, objections raised and dissipated, all these tend to fix in our minds the topics treated of, and prepare us for their more calm and rigid examination in our retirement. If the views advanced are valuable, if the argument is clear and logical, if the results announced are of practical worth, we have gained. If upon scrutiny, they do not appear in our judgment to be of very great value, yet have we gained; for the mental effort expended in their examination is beneficial; and besides this, the close contemplation of them will certainly inspire ideas of our own upon the same topics, which we can test and adopt or reject, "ever holding fast that which is good."

The third means of improvement which the twelve months of another year have given us is, the opportunity of putting in practice what we have learned.

The members of this Society are mostly, if not entirely, practicing Dentists. Daily are they brought into contact with cases requiring their professional services. These cases are as varied as the phases of human suffering. What a means of improvement have we here!

Theoretical knowledge is valuable only as it fits one for the practical duties of life. Having gained information by study, by examination, by oral teaching, it only remains for us to put this knowledge to its highest test by applying it to such actual cases as call for its exercise.

New remedies for certain diseases, new methods of manipulation, of treatment; new possibilities of successful operation which have come to our knowledge, inspire us with new confidence in our battle with the powers of destruction.

We apply the newly learned remedies, we employ the newly suggested methods, and their effect confirms our faith in them if they are good, or shows us their worthlessness. But dull indeed must be our minds if here, too, we fail to get new light from either our success or failure in their use. If success follows our effort we have gained a step, and the gain prompts to increased effort and successive steps of advancement. If we fail, we think, or *should* think, the harder.

It is by antagonism we grow. Failure is often the best teacher.

A more careful and minute scrutiny of the case, a more thorough use of instruments, a more critical investigation of the causes of failure, and a more thoughtful application of the chosen remedy;—these are its lessons, and wise ones they are. And though these are the fruits of a failure, they are, to the faithful observer of them, followed eventually by success.

"Reduce to practice all you learn," is the oft repeated injunction to the student, and it is no less applicable to established practitioners. Attack more and more difficult cases. Plant yourself firmly between the powers of destruction and the organ attacked and say, "thus far shalt thou come, but no farther." Then work to make your declaration true. Yield in no contest with disease until every known remedy is exhausted, and *then study for more.* Conquest must be added to conquest in this warfare. Our armor must not be laid aside until with it, we yield the principle that makes us living men.

Each victory has its double result also: satisfaction that we have achieved success, and greater strength for the next encounter.

Thus can we grow, if we honestly employ the *means* afforded us.

One great encouragement we have is found in the gigantic strides which Dentistry has taken during the past twenty—yes *ten* years.

Search them out if they are not familiar. Among my hearers, however, there can be none who are ignorant of them. The proof is found in every respectable Dental office, and in hundreds, yea thousands of the mouths of men.

The inducements to effort are numerous. Among noble men the gratification of a lofty ambition is sufficient. The desire for an extended reputation and the praise of men is another inducement, powerful among a certain class. And still another, if I must appeal to so low a motive, is self-interest.

No labor expended in fitting ourselves the better for benefitting the race, goes without its appropriate reward. Nor should it. "The laborer is worthy of his hire," was spoken eighteen hundred years ago, and is as true to-day, as then. Every man who works in the department of manual or mental labor, is entitled to just compensation. And if a man is not impelled to application by an inward ambition to excel, or by the desire of widespread reputation; scoff not at him if he yields to the motive of self-interest. It is a powerful motive, and few, very few, can be found who are not governed by it in some form.

We have not reached a period yet when the majority of men work merely from a love of hard work. Business is pursued for its product, and if by our organization, we are capable of experiencing enjoyment in the performance of faithful labor, in addition to the pleasure of receiving its more tangible fruit, happy are we.

Pursuing the thought of self-interest as a motive to exertion, it may be added.

Competency brings patronage. It is a false idea that the public do not appreciate eminent attainments. And yet many hold such an idea. It is true, however, that many so-called *dentists* do not appreciate eminent attainments. Indeed, some men pretend positively to despise them.

I have heard a Dentist ridicule his assistant because he used the name condyle instead of knuckle. It was denominated "putting on airs." Such Dentists know not the difference between a profession and a trade. They call their cases "jobs," their patients "customers," their laboratories "shops.' They scoff at *study* as necessary to fitness, and deem a skillful handling of tools in the "shop," as the best evidence of competency. It is not to be expected that such men will avail themselves of the advantages afforded by Dental societies or associations. These are not "in their way." It would be more in harmony with their views, to hang out strings of artificial sets of teeth from their windows, to be cut off and taken away by passing purchasers, as shoes from a store window, could it be done. It is not presumed that any of this class of Dentists ever come to the meetings of this Society.

Having noticed the three advantages which the past year has afforded us, viz: time, opportunity and means of improvement, and suggested inquiries of a practical character, the careful consideration of which, each one for himself, will supply the answer to the second main interrogatory—what have we done for ourselves? it only remains for me, gentlemen, to notice in brief, some of the instructive operations of this Society during the past year.

The following subjects have been discussed.

Filling the Roots of Teeth. Paper by Dr. Varney.

Physiology of the Blood. Paper by Dr. Atkinson.
Pathology of the Blood. Paper by Dr. John Allen.
Taking Impressions. Paper by Dr. Grout.
New Things observed during Vacation. Paper by Dr. Atkinson.
Foods and their effect. No paper.
Facial Neuralgia. No paper.
Peridental Inflammation. Paper by Dr. C. E. Latimer.
Extracting Teeth. Paper by Dr. Fiske.
Causes of Dental Caries. Paper by Dr. Weeks.
Cases for Artificial Teeth. Volunteer Paper by Dr. Weeks.
Bleaching Teeth. No paper.
Also a Volunteer Paper on Filling Roots of Teeth, by Dr. C. E. Latimer.

During the year, Dr. Wolworth of New Haven, visited the Society, and read a paper entitled "What next?" The subject of bread-making has also occupied the attention of the Society, having been introduced by a visitor, and snbsequently treated of by Dr. Weeks, one of our own members.

In conclusion, gentlemen, let me ask the question, how much has this Society done toward aiding its members in reaping benefit from the three advantages which the year has bestowed? For it will be observed that I have considered the *year* as a donor of these things, independent of any and all organizations.

Has this Society made a proper use of *its* opportunity to extend opportunities of improvement to its members?

Has *it* furnished any of the *means* with which the year has been laden?

A faithful answer to these questions will afford the proof of how much value a membership in this organization is possessed. If it has not, upon whom rests the blame? The Society works only through its members. Each one, therefore, in his measure, must bear the responsibility. If it has, whose is the credit? Theirs, and theirs alone, who have been faithful. Each one knows; and there I leave the matter.

Gentlemen, since I took this position, to which your confidence elevated me, we have advanced one stage on life's journey. The past of our lives is increased by the addition of twelve months of time, the future is reduced by the same period. What we have gained of wisdom, of experience, of knowledge, is so much increase of capital upon which to work through the coming year. Let us commence this new epoch with new energy, with firmer determination, with more serious thoughtfulness.

Time unimproved, swiftly leaves us; opportunities unseized, are lost for ever; means unused, become witnesses against us.

Oh, gentlemen, life is too short to be wasted; there is too much work to be done to be idle; we know too little, to neglect the smallest opportunity of acquiring knowledge. Let us be earnest, let us be diligent.

Gentlemen, my duties as President of this Society are nearly ended. When I have welcomed my successor to this chair, my last official act will have been performed.

Whatever of fault you have seen in me, forgive; whatever of good, remember. We have labored pleasantly and harmoniously together, and in yielding this position, let me express my hope that the future of this Society may be brighter than the past; that the spirit of harmony, without which there can be no prosperity, may dwell among you; that your course in the paths of scientific achievement may be onward and upward; that your history may be brilliant with noble accomplishments, distinguished for consistency and virtue, and such as shall deserve and command the admiration and respect the age.

*March* 11, 1868.

